# How to measure quality of surgery as a component of multimodality treatment of gastric cancer

**DOI:** 10.1002/ags3.12833

**Published:** 2024-06-12

**Authors:** Wojciech P. Polkowski, Katarzyna Gęca, Magdalena Skórzewska

**Affiliations:** ^1^ Department of Surgical Oncology of the Medical University of Lublin Uniwersytecki Szpital Kliniczny Nr 1 Lublin Poland

**Keywords:** complications, gastrectomy, gastric cancer, multimodality treatment, outcome, quality of life

## Abstract

Gastric cancer (GC) is one of the most frequent reasons for cancer‐related death worldwide. The multimodal therapeutic strategies are now pragmatically tailored to each patient, especially in advanced GC. A radical but safe gastrectomy remains the cornerstone of the GC treatment. Moreover, the quality‐of‐life (QoL) outcome measures are now routinely utilized in order to select optimal type of gastrectomy, as well as reconstruction method. Postoperative complications are frequent, and effective diagnosis and treatment of complications is crucial to lower the mortality rates. The postoperative complications prolong hospital stay and may result in poor QoL, thus eliminating the completion of perioperative adjuvant therapy. Therefore, avoiding morbidity is not only relevant for the immediate postoperative course, but can also affect long‐term oncological outcome. Measuring outcome enables surgeons to: monitor their own results; compare quality of treatment between centres; facilitate improvement both for surgery alone and combined treatment; select optimal procedure for an individual patient. Textbook oncological outcome is a composite quality measure representing the ideal hospitalization for gastrectomy, as well as stage‐appropriate (perioperative) adjuvant chemotherapy. Standardized system for recording complications and adherence to multimodality treatment guidelines are crucial for achieving the ultimate goal of surgical quality‐improvement that can benefit patients QoL and long‐term outcomes after fast and uneventful hospitalization for gastrectomy.

## Graphical abstract

The multimodal therapy is used for advanced gastric cancer. A radical gastrectomy remains the cornerstone of the treatment. Standardized system for recording complications and adherence to multimodality treatment guidelines are crucial to improve surgical quality that can benefit patients’ quality of life and long‐term outcomes.

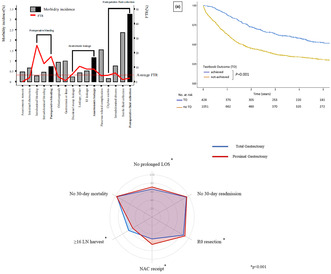

## 1 INTRODUCTION

Gastric cancer (GC) ranks among the most prevalent causes of cancer‐related death worldwide. GC is classified as the fifth most frequently encountered cancer and the fourth most lethal in terms of tumor‐related deaths among all types of cancers. Notwithstanding the worldwide decline in the prevalence and fatality of GC, recent cancer statistics demonstrate that there were 1 million new cases of GC and 76 900 deaths attributed to GC in 2020, with a male‐to‐female gender ratio of approximately 2:1.^1^

The mortality data can be attributed to a delay in diagnosis. The implementation of screening programs is exclusively limited to Asia, reflecting the incidence rates in the region. In Japan and South Korea, the utilization of endoscopy for GC has yielded a high rate of early disease detection. In contrast, in Europe and in the United States, the majority of GC are discovered with late symptoms in advanced stages. A gastrectomy involving D2 nodal dissection serves as the principal therapeutic approach for resectable GC, but overall 5‐year survival remains low at 30%–40%.^2^

Each patient is now provided with multimodal therapeutic strategies that have been pragmatically adjusted to their unique requirements. The surgical treatment is combined with perioperative (West) or adjuvant (East) chemotherapy (CTH) in locally advanced disease.^3^ The cornerstone of GC treatment continues to be a radical yet safe gastrectomy. Use of neoadjuvant radiation in addition to perioperative CTH for resectable GC should be made as part of a multidisciplinary discussion. Patients with tumors located at the esophago‐gastric junction seem to benefit most, as well as patients with a positive margin or inadequate lymphadenectomy.^4^ The ongoing clinical trials are expected to bring about changes in the timing and scope of curative resection, as the roles of CTH, radiation therapy, targeted molecular agents, and immunotherapies become clearer and better defined.^3^, ^5^

There is an unquestionable need to develop indicators for the assessment of the treatment quality, its impact on patients' quality of life (QoL), and the long‐term outcomes. This review will address the question of how to measure quality of surgery as a component of the multimodality treatment of GC.

## 2 NUMBER OF CANCER SURGERIES PERFORMED

An observed correlation has been found between low hospital surgery volume and higher rates of mortality and complications in multiple cancer types, including GC. The National Surgical Quality Improvement Program (NSQIP), which was established by the American College of Surgeons, is a nationally recognized and validated methodology that centres on measuring and improving the quality of surgical care by utilizing risk‐adjusted outcomes. It employs a prospective, peer‐controlled, validated database to quantify 30‐day, risk‐adjusted surgical outcomes, which provide a valid comparison of outcomes among all hospitals in the program. Peer‐reviewed studies have confirmed the effectiveness of NSQIP in enhancing the quality of surgical care, as well as reducing complications and costs.^6^

Patients who undergo high‐risk surgery at a hospital and under the care of a surgeon with extensive experience in the procedure achieve improved outcomes. Nevertheless, the establishment of minimum hospital volumes and minimum surgeon volumes for gastrectomy remains a topic of ongoing debate. In Japan, Iwatsuki et al. provided definitions for hospital and surgeon volumes pertaining to distal gastrectomy (DG) and total gastrectomy (TG).^7^ A threshold of over 52 and 51 cases per year has been designated for high hospital and surgeon volumes in DG. The minimum required volumes for TG are set at 27 and 26, respectively.^8^

In 2019, the German Cancer Society determined that the minimum caseload for gastric resections should be 20.^9^ The occurrence of in‐hospital mortality is lower in hospitals with a high volume of surgeries (50 per year) compared to hospitals with a low volume. The effectiveness of this outcome seems to hinge on the capacity to provide assistance to patients facing complications.^10^

Patients who underwent GC surgery at hospitals with higher case volumes experienced improved outcomes and reduced rates of failure to rescue (FtR) in the face of severe complications. According to a German analysis of patients who underwent GC resection between 2009 and 2017 in hospitals with an annual volume of over 30 resections, the mortality rate fell below 4%.^11^

As the surgeon's caseload reaches 30 cases per year, the mortality rate following resections for esophageal, gastric, and pancreatic cancer declines.^12^

The relationship between increasing surgeon proficiency and survival after gastric resection is obvious. A Scandinavian study documented a learning curve that led to improved long‐term survival after 20 supervised gastrectomies.^13^ Hence, it is imperative for surgeons to receive support and supervision throughout their initial 20 gastrectomies.

## 3 MORBIDITY

The consistent and comprehensive reporting of complications, an objective grading system that ranks complications based on severity, and the standardization of reporting are crucial for evaluating surgical quality.^14^ The Clavien–Dindo classification (CDC) is the most commonly utilized system for reporting complications, as it allows for the grading of complications based on their severity. Its expansion is known as the Comprehensive Complication Index (CCI). Additionally, it offers a metric approach to quantifying morbidity. The CCI demonstrates a more pronounced correlation with postoperative hospital stay in comparison to the conventional CDC.^15^ Nevertheless, according to data from the LOGICA‐trial, the implementation of the CCI did not demonstrate any clinically significant advantages and instead resulted in increased workload compared to the CDC for evaluating the complication burden.^16^ The complication monitoring model, known as cumulative sum control‐CCI (CUSUM‐CCI), can be displayed for individual surgeons, as well as for making comparisons between surgeons and the institutions.^15^

## 4 MORTALITY

The concept of failure to rescue (FtR) allows for the characterization of a delay or failure in the recognition and appropriate management of postoperative complications, frequently resulting in a fatal outcome. The FtR favors centers that have a large volume and a continuous presence of interdisciplinary experts.

The relationship between the FtR and caseload is interdependent. As demonstrated in German experience, there is a correlation between high surgical volume and improved patient outcome. This correlation is determined by the yearly number of resections certified by the DKG as a categorical indicator of high surgical volume and in‐house mortality as an indicator of patient outcome. The fulfillment of the annual surgical minimum for gastrectomy was found to be correlated with a decrease in in‐house mortality and FtR rates in cases involving complications.^17^

**FIGURE 1:**
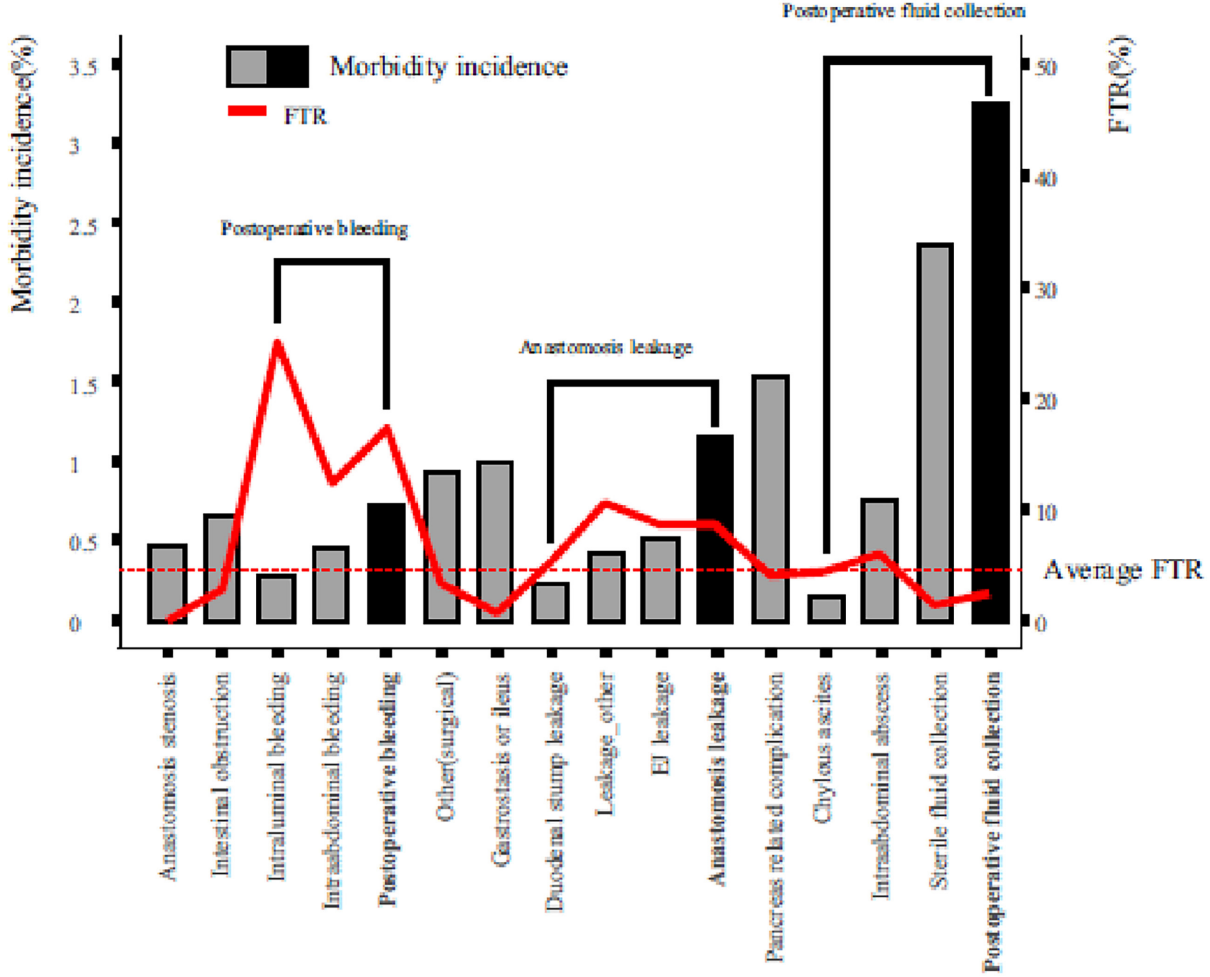
The red line indicates the FtR rate and is displayed on the right y‐axis. Surgical morbidity incidence is displayed on the left y‐axis. With permission from Park et al., Changes in failure to rescue after gastrectomy at a large‐volume center with a 16‐year experience in Korea.^18^

The identification of risk factors for FtR has been achieved through a large‐scale comprehensive study conducted in a high‐volume Korean center. These risk factors include old age, high ASA score, serosa exposure, and operation period from 2006 to 2021.^18^ The most frequent and fatal systemic complications were acute respiratory distress syndrome, coronary artery disease, renal complications, and stroke. The graphical representation (Figure 1) effectively presents the incidence of morbidity and FtR rate based on different types of surgical complications.

Surgical treatment should be administered cautiously to patients who are predicted to have a high FtR rate. The FtR rate was not influenced by the operation method or resection extent in the presence of complications.

## 5 THOROUGH REPORTING OF COMPLICATIONS

The reporting of complications can be achieved on a (inter‐) national level by adhering to internationally accepted definitions. The Esophagectomy Complications Consensus Group (ECCG) has created a standardized outcomes set to ensure consistent reporting of outcomes in esophageal surgery.^19^ The Dutch Upper gastrointestinal Cancer Audit DUCA outlines that complications following gastrectomy were observed in 43% of patients overall, with 19% of patients experiencing major complications (CDC grade III or higher).^20^ In 2015, the European Chapter of the International Gastric Cancer Association (IGCA) commenced a project with the primary goal of formulating a list and precise definitions of postoperative complications that are specific to gastrectomy.^21^ A multicenter observational study was conducted to validate the GASTRODATA list of complications, encompassing all consecutive resections conducted over a 2‐year period.^22^ The overall morbidity rate was recorded at 29.8%. The median CDC and CCI scores were IIIa and 26.2, respectively. In‐hospital, 30‐day, and 90‐day mortality rates were 3.2%, 3.6%, and 4.5%, respectively. Through the European Registration of Cancer Care (EURECCA) Upper GI project, a core dataset was formed comprising of 46 shared items,^23^ and discrepancies in the approach to GC treatment and patient survival rates in several European countries were unveiled.^24^, ^25^ The application of predefined standards in reporting outcomes following major upper gastro‐intestinal surgery (ECCG) allows for the comparison of complication rates from existing sources (DUCA) with those of an unselected international cohort from the Oesophago‐Gastric Anastomosis Audit (OGAA).^26^ In the time frame of 2015–2019, the Italian Research Group for Gastric Cancer (GIRCG) has endorsed a compilation of complicated gastrectomy cases (*n* = 386, mean 1.4 complication/patient).^27^ The study suggests that the standardized set of complications and registry created by the International Gastrectomy Complications Database may be employed for outcome reporting and audit on a national scale.

## 6 COMPOSITE SURGICAL QUALITY MEASURES

The term “textbook outcome” (TO) was initially employed in 2013 to provide a qualitative evaluation of the efficacy of surgical intervention in patients diagnosed with colorectal cancer.^28^ The TO is a novel composite quality measure that encompasses various postoperative endpoints, exemplifying the ideal hospitalization for complex surgical procedures. Regarding gastrectomy, it comprises the subsequent characteristics, as initially outlined by the DUCA:

1. Resection margin (macroscopically)
2. Resection margin (microscopically)
3. Number of retrieved lymph nodes ≥15
4. Intraoperative complications
5. Postoperative complications
6. Re‐interventions
7. Admission to ICU
8. Prolonged hospital stay (>21 days)
9. Readmission
10. 30‐day mortality.^29^

TO describes a portion of patients who underwent high‐quality smooth surgery with an optimal postoperative course. Textbook oncological outcome (TOO) encompasses not only skillful surgery with uncomplicated intra‐ and postoperative course but also the appropriate administration of perioperative chemotherapy (POC) for GC in accordance with guidelines.

We have put forth a proposal to augment the existing definition of TO by incorporating an additional characteristic, which is compliance with POC. The TOO is associated with improved survival. It could potentially function as a quality parameter for multimodal treatment in patients with advanced GC.^30^

Table 1 displays the variations in the published definitions of TO and TOO.

**TABLE 1:** Comparison of TO/TOO definition in publications on GC.

Author/year	Radicality according to surgeon	No intraoperative complications	R0 resection	>15 LN retrieved and examined	No severe postoperative complications	No re‐interventions	No ICU	No prolonged LOS	No postoperative mortality	No re‐admission	POC receipt of guideline‐concordant chemotherapy	TO (%)	TOO (%)
Busweiler et al. 2017^a^, ^32^	+	+	+	+	+		+	+	+	+		32.1	
van der Kaaij et al. 2018^a^	+	+	+	+	+	+	+	+	+	+		45.7	
van der Werf et al. 2019^a^	+	+	+	+	+	+	+	+	+	+		35.0	
Levy et al. 2020 & 2022^45^, ^46^			+	+	+	+	+	+	+	+		22.8	
Priego et al. 2019^47^			+	+	+					+		51.0	
Aquina et al. 2021^48^			+	+				+		+	+		31.8
Roh et al. 2021^49^		+	+	+	+	+	+	+	+	+		61.5	
Sędłak et al. 2021^30^	+	+	+	+	+	+	+	+	+	+	+		40.2^b^
Spolverato et al. 2021^50^			+	+				+	+		+		35.3
Bolger et al. 2021^a^	+	+	+	+	+	+	+	+	+	+		37.6	
Voeten et al. 2021^52^		+	+	+	+		+	+	+	+		54.2	
Chen et al. 2022^53^	+	+		+	+	+	+	+	+	+		84.2	
Cibulas et al. 2022^54^			+	+				+		+	+		23.8
Dal Cero et al. 2022^55^	+		+	+	+	+		+	+	+		41.1	
Kamarajah et al. 2022^56^			+	+				+	+	+		41.4	
Carbonell Morote et al. 2023^57^			+	+	+			+	+	+		34.1	
Sędłak et al. 2023^58^	+	+	+	+	+	+	+	+	+	+	+	68.5	22.8^c^

Abbreviations: ICU, intensive care unit; LN, lymph nodes; LOS, length of stay; POC, perioperative chemotherapy; TO, textbook outcome; TOO, textbook oncological outcome.

^a^ DUCA definition.

^b^ Single center.

^c^ Multicenter data.

Despite the continuous improvement in surgical outcomes, adherence to current guidelines for stage‐appropriate CTH in the multimodal treatment of GC remains inadequate. Further enhancement of oncologic quality metrics necessitates a heightened focus on perioperative therapy and ensuring the adequacy of lymphadenectomy.

**FIGURE 2:**
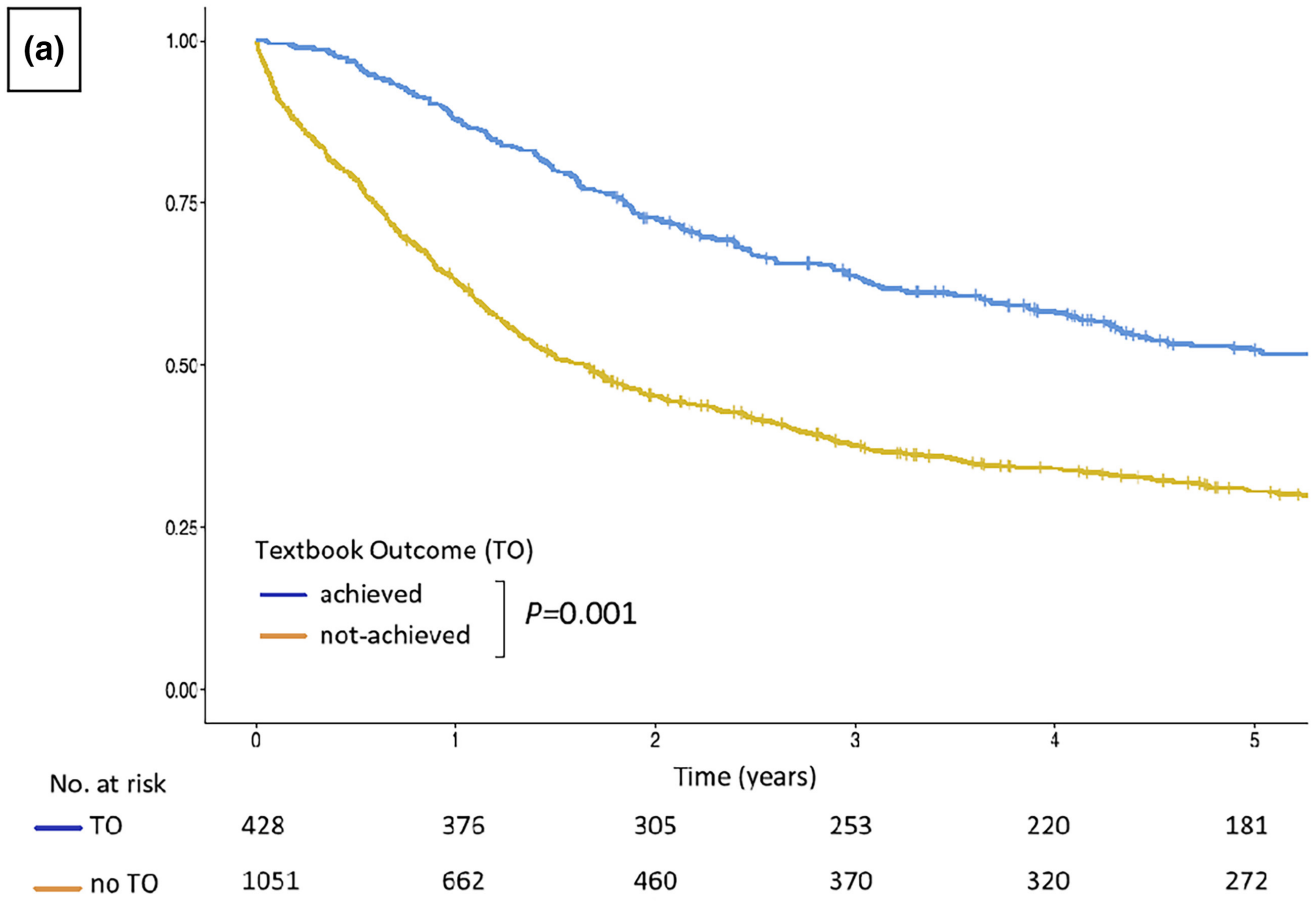
Kaplan–Meier survival curves for overall survival of patients who underwent surgery for gastric cancer for groups with and without a Textbook Outcome (TO) (log rank test). With permission from Bobrzynski et al., Evaluation of optimum classification measures used to define textbook outcome among patients undergoing curative‐intent resection of gastric cancer.^31^

A uniform definition of TO provides clinically relevant prognostic information and could be used for surgical quality improvement in GC patients. In order to achieve that objective, we conducted a review of prospectively maintained electronic databases. These databases included information from 1743 patients who were treated in two academic surgical centres in Poland.^31^ A total of six candidate definitions of TO were assessed in terms of their capacity to accurately forecast patients' prognosis. The combination of 10 measures, which correspond to complete tumor resection and an uneventful postoperative course, yielded the best goodness of fit and predictive performance in defining TO. The overall median survival was significantly longer for patients with than without TO (69 vs. 20 months; Figure 2). TO retained its prognostic significance in a multivariate model, even after adjusting for age, sex, comorbidities, treatment, and tumor‐related variables. Furthermore, it was linked to a 39% decrease in the risk of mortality.

Conversely, the introduction of the TO index aims to quantify the proportion of patients who undergo surgery and subsequently experience an optimal postoperative course. The definition of TO can encompass various parameters, including the absence of intraoperative complications, achieving a free tumor resection margin, appropriate identification of lymph nodes in the resected specimen, and the absence of severe postoperative complications in GC surgery.^32^ In heterogeneous patient groups, it is crucial to acknowledge that the use of TO may not yield fair comparisons between institutions or countries. In that sense, comparison of outcomes in the best patient set is necessary.

## 7 BENCHMARKING

Benchmark values have already been established for numerous procedures within diverse fields of gastro‐intestinal surgery.^14^ One potential application of the benchmark values is to identify patients with outcome parameters that fall outside of the benchmark range, for the purpose of discussing their cases at institutional, multidisciplinary morbidity‐mortality conferences. A potential explanation for outcomes that are inferior to benchmark values could be attributed to inadequacies in the provision of care, or simply due to the fact that the patient belongs to a higher risk category. Most benchmark studies have identified a new finding in determining the best centres. In addition to the well‐established center volume or rates of FtR, centers of excellence disclosed a high proportion of higher risk patients (i.e., non‐benchmark patients). Consequently, the ratio between benchmark and non‐benchmark cases establishes a novel surrogate marker of quality.

The prerequisite condition for the proper use of benchmark metrics for comparisons, however, is the accurate data collection. The first study to provide benchmark values for outcomes after gastrectomy collected data from a large group of patients treated in expert centres from various countries, including the European GASTRODATA consortium. By analyzing a subgroup of “ideal” patients with low comorbidity, “best possible” results for different types of oncological gastrectomy were obtained.^33^

## 8 PATIENT‐REPORTED OUTCOME

The patient‐reported outcome measures (PROMs) and patient‐reported experience measures (PREMs) provide the most reliable indication of the actual impact of surgery on a patient's life and satisfaction. The PROMs include questionnaires completed by patients without professional interference, using paper or electronic applications; generic with questions related to pain, general quality of life, social impact, or condition, including questions focused on disease symptoms or consequences of the surgical procedure. The PREMs comprise surveys completed by patients to assess their experiences and include questions related to communication with healthcare providers, timeliness of care, and overall satisfaction with the care provided.^14^

Health‐Related Quality of Life (HR‐QoL) measurements, encompassing physical, social, and psychological aspects, are crucial in healthcare.^34^ The EQ‐5D‐5L has been identified as an optimal tool for assessing these dimensions, with suggestions for additional modules to improve its effectiveness.

The Patient‐Reported Outcomes Measurement Information System (PROMIS) is a promising tool in this area, offering a novel approach with its Item Response Theory (IRT)‐based item banks.^35^ Despite its potential, PROMIS is underutilized in GC studies, particularly in non‐bariatric patients post gastrectomy, indicating a need for more comprehensive research and application in this patient group.^36^

Further insights come from a meta‐analysis by van den Boorn et al., highlighting that the most substantial decline in global health status occurs within the first month post gastrectomy, with a return to baseline levels after 12 months.^37^ Interestingly, this analysis found that while esophagectomy significantly affects HR‐QoL, gastrectomy does not. Additionally, the European STOMACH trial found that minimally invasive gastrectomy led to higher initial pain and dysphagia scores, which normalized after 6 months. This trial, involving 96 patients treated with either open or minimally invasive total gastrectomy post neoadjuvant chemotherapy, did not find significant differences in HR‐QoL between the two surgical methods after 1 year.^38^

## 9 POSTOPERATIVE QUALITY OF LIFE (QOL)

Lack of a suitable instrument to comprehensively assess symptoms, living status, and QoL in surgical GC patients prompted the Japanese Postgastrectomy Syndrome Working Party to develop postgastrectomy syndrome assessment scale (PGSAS)‐45. The prevalence of postgastrectomy syndrome and its impact on daily life among patients who underwent different types of gastrectomy was determined through a multi‐institutional survey using the PGSAS‐45. The administration of TG and proximal gastrectomy (PG) resulted in a significant decline in the postoperative QoL of patients. The gastrectomy procedure resulted in significant differences among the surgical procedures in terms of the most noticeable adverse effects, namely the meal‐related distress subscale, dissatisfaction at the meal, and weight loss. The PGSAS‐45 scores after the six main gastrectomy procedures revealed that the postoperative QoL differed greatly depending on the site and extent of gastrectomy. In the selection of gastrectomy procedures, it is crucial to consider the severity and characteristics of postgastrectomy syndrome as a means to overcome surgical shortcomings and improve postoperative care.

A comprehensive analysis of the PGSAS‐NEXT study reveals the identification of several influential factors on the main outcome measures post‐surgery for proximal GC. Multiple regression analysis of main outcome measures revealed that change in body weight, necessity for additional meals, ability to work, and dissatisfaction with working, have been found to be significantly better after PG than after TG. Therefore, it is advisable to consider performing PG for proximal tumors, even in cases of advanced cancer, in order to achieve a favorable postoperative QoL, provided that oncological safety is ensured.

A steady increase in gastroesophageal junction and proximal GC incidence has been observed in the West. The translation of Japanese QoL data into Western surgical treatment of patients with advanced proximal GC can potentially enhance postoperative QoL. This can be achieved by opting for PG when no proximal part of the stomach can be preserved, or by constructing a jejunal pouch if TG is unavoidable. Given that QoL after TG is extremely poor, the question arises as to whether it is appropriate to consider an organ‐sparing approach for patients with proximal GC.

**FIGURE 3:**
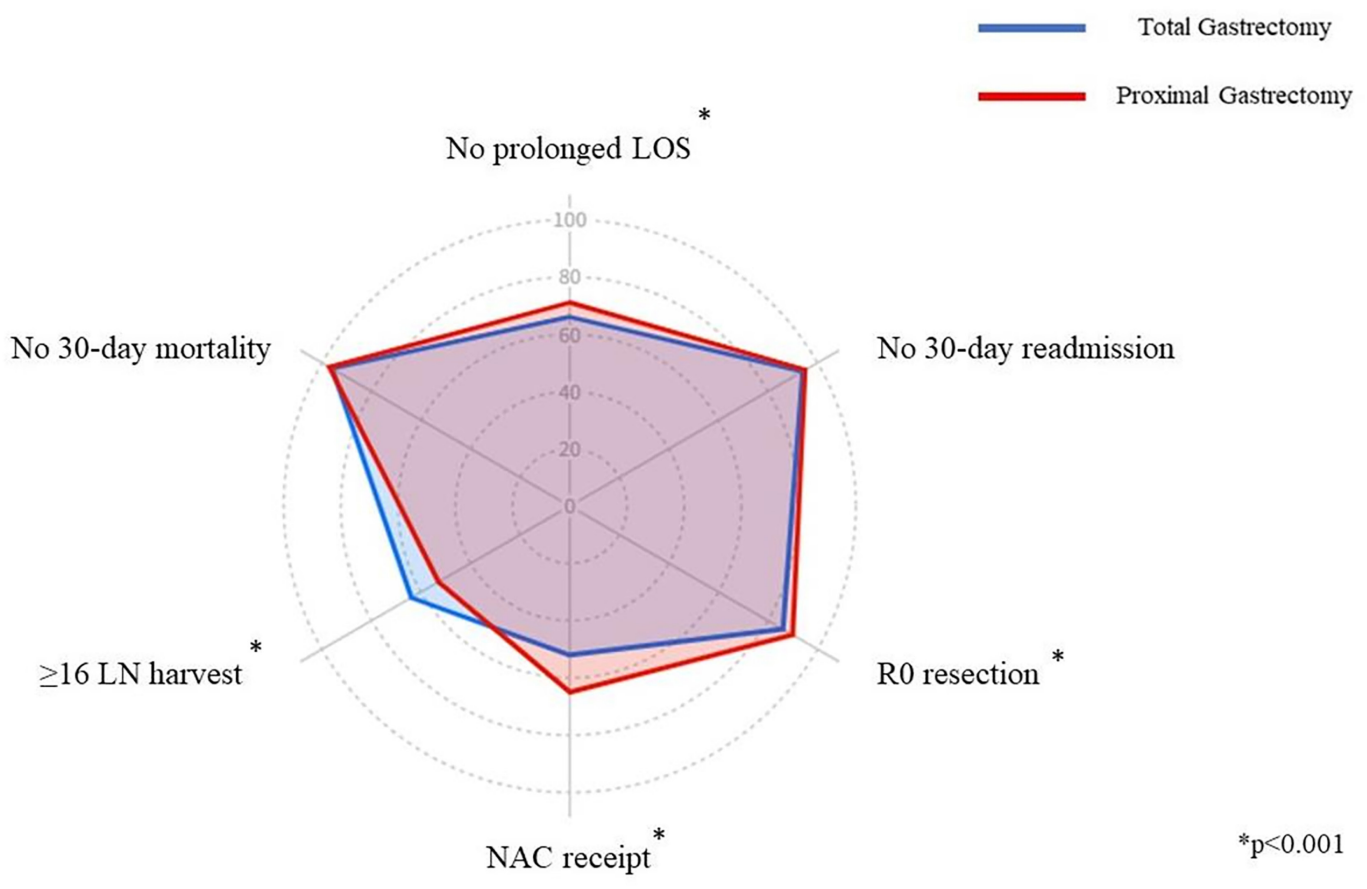
Assessment of TOO parameters in proximal GC patients undergoing TG and PG. With permission from Rawicz‐Pruszyński et al., Proximal Gastric Cancer—Time for Organ Sparing Approach?^43^

The latest data from the United States provides sufficient justification for additional research in this field. Short‐ and long‐term outcomes of 7616 patients with stage II/III proximal GC who underwent TG or PG with curative intent between 2009 and 2019 were analyzed based on the National Cancer Database (NCDB). The PG and TG were performed on 69% and 31% patients, respectively. TG patients displayed a higher incidence of pT4 tumors, metastatic lymph nodes, and evaluation of more than 16 lymph nodes. In contrast, they exhibited a lower incidence of negative resection margins. Figure 3 displays the comparison of outcome measures after TG versus PG.

The consensus conference provided recommendations regarding the assessment of outcomes for surgical interventions.^39^ The Jury consisted of independent members, including key stakeholders from various sectors such as economy, industry, psychology/psychiatry, science, and patient advocates. The Outcome for Medicine Consensus Conference (Jury recommendations) concluded with seven final statements.

1. Record outcome parameters at standardized time points
2. Routinely use PROMS and PREMS in clinical care
3. Record individual and global morbidity according to the CDC and by using the CCI
4. Define benchmark values and compare results
5. Conduct routine interdisciplinary mortality and morbidity conferences
6. Appoint a “data quality guarantor” at every institution
7. Follow the TRACK principle in case of unwarranted outcomes: Transparency, Respect, Accountability, Continuity, and Kindness must be applied.

The role of a surgeon should be more clearly delineated as that of a patient expectation manager in the context of a tailored patient approach that commences prior to the surgical journey. An excellent illustration of such research involves an endeavor to forecast QoL, both in the short and long term, for patients with esophageal cancer following neoadjuvant CTH and esophagectomy.^40^ Following this multimodality treatment, patients in a favorable condition show a more significant decrease in health‐related QoL. Understanding the expectations of patients prior to treatment is of utmost importance.

The integration of big data in surgical quality control has been revolutionized by databases such as the SEER program in the United States and Japan's National Clinical Database (NCD). These databases provide extensive data on cancer statistics and surgical outcomes, enabling healthcare professionals to benchmark performance and implement evidence‐based improvements. They signify a shift towards a data‐driven approach in surgical quality control, highlighting the growing importance of big data in healthcare. The National Cancer Data Base (NCDB) in the United States collects comprehensive data on cancer cases and supports quality improvement at the local level through web‐based audit and feedback reporting tools. However, there is a need for validation of the data reported to ensure accuracy and completeness. The NCDB manages data quality control through the use of software and collaboration with other organizations.^36^, ^41^, ^42^

## 10 CONCLUSIONS

The establishment of a standardized system to record complications and adherence to multimodality treatment guidelines is of utmost importance in order to achieve the ultimate objective of enhancing surgical quality. This, in turn, can have a positive impact on QoL and long‐term outcomes for patients with advanced GC. By employing this standardized system, it would be possible to ensure the consistent and precise monitoring of complications, as well as the adherence to treatment guidelines, such as stage/R‐appropriate chemo‐ and/or radiotherapy. Through the implementation of a standardized system, healthcare providers can identify areas of improvement, assess the efficacy of interventions, and make data‐driven decisions to enhance patient care. Furthermore, the effectiveness of gastrectomy can be assessed by employing a composite quality measure known as TOO. This measure considers various factors, such as surgical technique, perioperative care, and the utilization of systemic treatment. Hospitals can secure the highest standard of care for patients by striving for excellence of TOO, leading to enhanced survival, QoL, and long‐term outcomes of patients with advanced GC.

## CONFLICT OF INTEREST STATEMENT

Authors declare no conflict of interests for this article.
